# Modeling choice and reaction time during arbitrary visuomotor learning through the coordination of adaptive working memory and reinforcement learning

**DOI:** 10.3389/fnbeh.2015.00225

**Published:** 2015-08-26

**Authors:** Guillaume Viejo, Mehdi Khamassi, Andrea Brovelli, Benoît Girard

**Affiliations:** ^1^Sorbonne Université, Université Pierre et Marie Curie, Univ Paris 06, UMR 7222, Institut des Systèmes Intelligents et de RobotiqueParis, France; ^2^Centre National de la Recherche Scientifique, UMR 7222, ISIRParis, France; ^3^Institut de Neurosciences de la Timone, UMR 7289, Centre National de la Recherche Scientifique - Aix Marseille UniversitéMarseille, France

**Keywords:** behavior, action selection, decision-making, working-memory, reinforcement learning, reaction times, multi-objective optimization

## Abstract

Current learning theory provides a comprehensive description of how humans and other animals learn, and places behavioral flexibility and automaticity at heart of adaptive behaviors. However, the computations supporting the interactions between goal-directed and habitual decision-making systems are still poorly understood. Previous functional magnetic resonance imaging (fMRI) results suggest that the brain hosts complementary computations that may differentially support goal-directed and habitual processes in the form of a dynamical interplay rather than a serial recruitment of strategies. To better elucidate the computations underlying flexible behavior, we develop a dual-system computational model that can predict both performance (i.e., participants' choices) and modulations in reaction times during learning of a stimulus–response association task. The habitual system is modeled with a simple Q-Learning algorithm (QL). For the goal-directed system, we propose a new Bayesian Working Memory (BWM) model that searches for information in the history of previous trials in order to minimize Shannon entropy. We propose a model for QL and BWM coordination such that the expensive memory manipulation is under control of, among others, the level of convergence of the habitual learning. We test the ability of QL or BWM alone to explain human behavior, and compare them with the performance of model combinations, to highlight the need for such combinations to explain behavior. Two of the tested combination models are derived from the literature, and the latter being our new proposal. In conclusion, all subjects were better explained by model combinations, and the majority of them are explained by our new coordination proposal.

## 1. Introduction

Learning the consequence of actions and consolidating habitual responses are key cognitive functions because they embody behavioral flexibility and automaticity. Acquisition and consolidation of instrumental behavior are known to engage distinct decision-making processes. Acquisition relies on flexible goal-directed actions selected according to the expected outcomes as well as current goals and motivational state (Rescorla, 1991; Dickinson and Balleine, 1994; Staddon and Cerutti, 2003). Consolidation is characterized by the gradual formation of stimulus-driven habitual responses (Dickinson, 1985; Dickinson and Balleine, 1993). At the neural level, converging evidence from human neuroimaging and animal studies confirms a dual-system hypothesis underlying instrumental behaviors. Goal-directed actions are thought to be primarily controlled by the associative frontostriatal circuit including the lateral and medial prefrontal cortices and the caudate nucleus. Habitual motor responses are thought to recruit neural pathways, linking sensorimotor and premotor areas with the putamen (for reviews see Yin et al., 2006, 2008; Balleine et al., 2007; Graybiel, 2008; Packard, 2009; Ashby et al., 2010; Balleine and O'Doherty, 2010). Computational formulations of goal-directed and habitual processes model them as distinct and complementary reinforcement learning (RL) “strategies” (Doya, 1999; Daw et al., 2005; Redish et al., 2008; Dollé et al., 2010; Keramati et al., 2011; Collins and Frank, 2012; Khamassi and Humphries, 2012; Botvinick and Weinstein, 2014). The goal-directed system is thought to learn a model of the environment including action-outcome (A-O) contingencies and transition probabilities, and can, therefore, be formalized using “model-based” RL algorithms. Instead, the habit system is thought to learn stimulus–responses relations by reinforcing successful behaviors through reward-prediction error signals without creating internal models of the environment, using “model-free” RL algorithms. Accordingly, goal-directed behaviors resulting from model-based RL strategies appear flexible (i.e., can adapt to changing A-O contingencies) but cognitively expensive (i.e., require inference of action outcomes and are potentially slow); habitual behaviors modeled using model-free RL strategies, however, are more stable (i.e., they are not sensitive to rapid changes in A-O contingencies) and are more rapid (i.e., the action with the highest value is chosen). In other words, a cognitive agent can use a complex internal structure of the world to make an accurate decision given a certain cost and decision-making time, or he can exploit a degraded and valued representation of the world that makes it quicker to decide but slower to adapt.

Neuroimaging evidence has accumulated over recent years, thereby supporting the existence of two complementary forms of learning signals each associated with different RL strategies. Neural correlates of state prediction-error signals (putative hallmark of “model-based” processes) have been found in the intraparietal sulcus and lateral PFC, in addition to reward prediction errors (putative hallmark of “model-free” processes) in the ventral striatum (Gläscher et al., 2010). In particular, the dorsal striatum has been shown to host complementary computations that may differentially support goal-directed and habitual processes (Brovelli et al., 2011): the activity of the anterior caudate nucleus correlated with the amount of working memory and cognitive control demands, whereas the putamen tracked how likely the conditioning stimuli lead to correct response. The dynamic interplay between goal-directed and habitual processes, rather than their serial recruitment, has been shown during learning to parallel portion of reaction times variance during learning (Brovelli et al., 2011). Driven by these insights, this work is focused on capturing, with computational tools, the specific behavioral results of this instrumental learning task (i.e., choices and reaction times). As suggested by the neuroimaging results, we build and compare models with the hypothesis that a dual-strategy system is required for this particular task.

In addition, another study has challenged the notion of non-overlapping neural substrates by showing that the ventral striatal BOLD signal of reward prediction errors, classical manifestation of model-free learning strategy, reflected also model-based predictions in proportions matching those that best explained choice behavior (Daw et al., 2011). More recently, BOLD signals in the inferior lateral prefrontal and frontopolar cortex have been found to be correlated with the reliability of the predictions of the model-based and model-free systems, respectively, therefore suggesting an arbitration mechanism allocating control over behavior based on reliability signals (Lee et al., 2014). Overall, neuroimaging studies suggest complex interactions between model-free and model-based systems, whose interplay and relation with choice accuracy and speed (i.e., reaction times) are partly elucidated.

Arbitration mechanisms have been formalized using different approaches. Daw et al. (2005) were the first to suggest how unified behavior emerges from the interaction between the goal-directed and habitual systems, and proposed an arbitration process based on the uncertainty in the model's estimates, such that the final choice is controlled by the system whose estimate of action values is the most accurate. Another study (Keramati et al., 2011) suggested that arbitration may rely on a speed/accuracy trade-off between the two systems and a nearly optimal balance between reaction-time and accuracy. By pulling the properties of each strategies to the extremes (perfect but slow model-based vs. potentially inaccurate and fast model-free), a trade-off between speed and accuracy can account for behavioral observations in rodents during instrumental learning. Such model correctly predicts differences in sensitivity to devaluation between moderate and extensive training. More recently, capacity-limited working memory processes have also been incorporated into the arbitration mechanisms and it has been shown to capture behavioral variance that could not be captured in a pure RL framework (Collins and Frank, 2012). These computational models have provided significant insight into the possible computations mediating arbitration. However, none provided a comprehensive account modeling both behavioral performance (i.e., choices) and speed-accuracy tradeoffs (i.e., reactions times) during the acquisition and stabilization of an instrumental task.

In this study, we address this issue by building on previous accounts of model-free and model-based learning. First, we propose a new model of Bayesian Working Memory (BWM) to account for goal-directed computations in sensorimotor learning tasks where subjects need to learn the sequence of previous choices and outcomes to deliberate about future choices (Brovelli et al., 2011; Enomoto and Matsumoto, 2011; Collins and Frank, 2012; Khamassi et al., 2015). This relatively high-level abstraction of the working memory processes is based on the Bayesian mathematical formalism. A recent study has already explored this level of formalism to predict the limited capacity of working memory (Morey, 2011). The tool of Shannon entropy is borrowed from Information theory. It has been shown to be fruitful to explain temporal variability in perceptual process (Norwich, 2003). Within the domain of this work, i.e., human decision-making, measures of entropy have been used to explain variations of activity in the prefrontal cortex (Koechlin and Hyafil, 2007). In this study, the entropy is heavily used as a measure of uncertainty computed upon probabilities of actions. In addition, we explore the idea of the entropy as a self-monitoring variable that measures the information gained from retrieving memory items. The habitual process is modeled using a Q-Learning algorithm (QL) (Watkins and Dayan, 1992).

Second, while most models of strategy selection tend to oppose models and choose concurrently goal-directed or habitual decisions according to uncertainty criteria, we propose an arbitration process which assumes that relevant information retained in working memory is selectively accessed during learning. Within this view, QL and BWM are combined such that memory manipulation is limited by, among other, the “strength” of habitual learning.

Once again, the entropy of action probabilities from both systems is used in order to dynamically control the working memory retrieval process. In order to compare with previous approaches, the proposed model is compared with previously proposed arbitration mechanisms. From Keramati et al. (2011), we adapted the speed-accuracy trade off, resulting in a pure selection mechanism (i.e., each strategy is selected concurrently). From Collins and Frank (2012), we derived a mixture model that weights each strategy in order to compute final probabilities of action.

Third, contrary to previous accounts modeling exclusively choice accuracy, we fitted model's parameters to both choice accuracy and reaction times using the NSGA-2 multi-objective evolutionary algorithm (Mouret and Doncieux, 2010). In particular, while the usefulness of dual-system learning models with a large number of parameters over single-system models is often difficult to prove statistically when fitting only choices using standard criteria such as BIC, fitting both choices and reaction times clearly stress the need for a dual-system learning model. We describe step-by-step the new multi-objective model comparison method to make it usable in different experiments and contexts.

We were, therefore, able to provide optimal estimates of the interplay between model-free and model-based strategies that leads to new interpretations of the dual-learning problematic. We show that the arbitration mechanism we propose is best suited to explain both choices and reaction times of the experimental results of Brovelli et al. (2011). We also predict the amount of information used to decide at trial step and these predictions show differences between each architecture of strategy selection.

## 2. Models

### 2.1. Arbitrary visuomotor learning task

Arbitrary visuomotor learning is defined as the ability to learn arbitrary and causal relations linking visual inputs to actions and outcomes (Wise and Murray, 2000). Previous fMRI studies have shown that both the processing of outcomes (Brovelli et al., 2008) and selection of action (Brovelli et al., 2011) during arbitrary visuomotor learning conform to neural computations predicted by instrumental learning theory. We, therefore, assume that arbitrary visuomotor learning tasks represents a canonical instance of instrumental learning and can be used to study the acquisition and early consolidation of instrumental behaviors, during which both the goal-directed and habitual systems are thought to coordinate. Once consolidated, arbitrary visuomotor mappings may form the basis of highly-acquainted instrumental behaviors, such as habits. Here, we tested the proposed computational model on behavioral data acquired from an arbitrary visuomotor learning task that required participants to learn by trial-and-error the correct association between three-colored circle and five-finger movements (Brovelli et al., 2008, 2011).

At each trial, a colored circle was presented to which the participant had to respond within 1.5 s. After a variable delay ranging from 4 to 12 s, a feedback image was presented to inform whether the selected motor response was correct or incorrect. The order of visual stimuli was randomized and subjects were specifically informed about the independence between stimuli, i.e., the correct response for one stimulus did not predict the correct response for other stimuli. Each participant performed four learning blocks each lasting 42 trials. To solve the task, subjects had to memorize previous response to avoid repeated errors, and once the correct associations were found, only these associations are worth memorizing.

**Figure 1:**
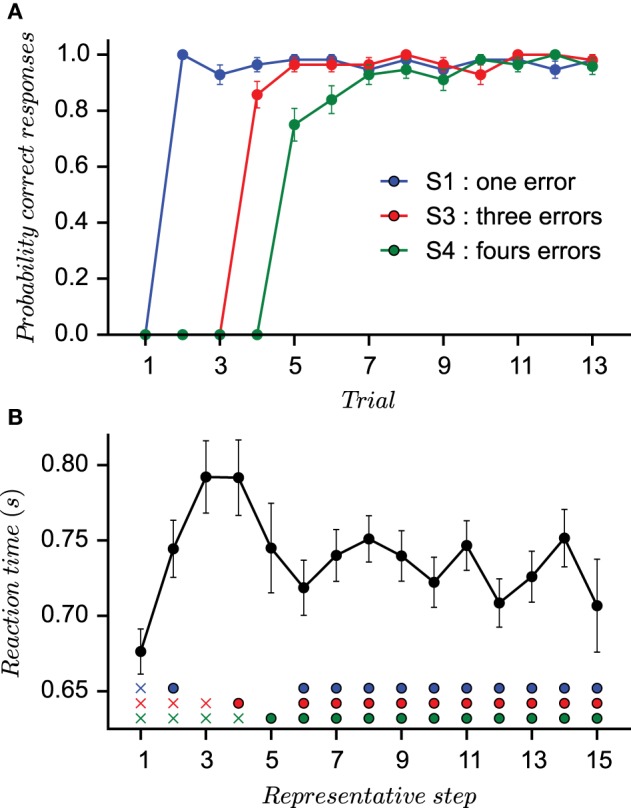
**Behavioral results**. **(A)** The three curves (one for each stimulus) representing the probability of correct response vs. the number of stimulus presentation (not according to the actual appearance during the learning session) computed on the sequence of outcomes (1 for correct and 0 for incorrect). The small standard error values provide evidence for quasi-stereotyped learning behaviors. **(B)** The mean reaction times of all subjects is computed after a reordering of individual reaction times according to representative steps (Brovelli et al., 2011). Rows of marks indicates the position of reaction times before the subject is given the right answer (crosses) and after (circles).

To ensure highly reproducible performances across sessions and subjects, the correct stimulus–response associations were not set *a priori*. Instead, they were assigned as subject proceed in the task. The first presentation of each stimulus was always followed by an incorrect outcome, irrespective of subject's choice. On the second presentation of stimulus S1, any new untried finger movement was considered as a correct response. For the second stimulus S3, the response was defined as correct only when the subject had performed three incorrect movements. For stimulus S4, the subject had to try four different finger movements before the correct response is found. In other words, the correct response was the second finger movement (different from the first tried response) for stimulus S1, the fourth finger movement for stimulus S3, and the fifth for stimulus S4. This task designs assured a minimum number of incorrect trials during acquisition (1 for S1, 3 for S3, and 4 for S4; the stimuli number is a direct reminder of the number of errors required) and fixed representative steps during learning. In particular, it produces highly reproducible behavioral performances across sessions and subjects as it can be observed from the small standard deviations evaluated from the probability of correct responses (PCR) for each stimulus (Figure 1A).

Reaction times (RT) were measured as the interval between the presentation of the stimulus and the button press. In order to visualize the evolution of mean reaction times (RTs) during the experiment, a reordering process was applied that provides a mean value over representative steps as in Brovelli et al. (2011) (see Section 5). The first five representative steps form the acquisition phase in which a subject does the required errors, whereas the next 10 representative trials constitute the consolidation phase. Mean RTs at the first representative step were averaged over the first incorrect response for each stimulus. Mean RTs at the second representative step were averaged over the correct response for stimulus S1 and second incorrect responses for stimuli S3 and S4. Each following steps (3–5) is an average over either the first correct responses or required incorrect responses. From Step 6 to 15, mean RTs were an average from the second presentation of the stimulus after the correct associations were given at the end of the session. RTs, therefore, were averaged over correct and incorrect responses since incorrect feedbacks occur after true association was given. As shown in Figure 1B, the evolution of RTs shows a characteristic pattern: RTs are short at the beginning of acquisition; they lengthen as errors accumulate when the decision process from Step 2 to 4 slows down since subjects must engage into cognitive processes to remember precious errors. Then, they decrease again when the correct responses are learned.

### 2.2. Computational models

To disentangle possible computational mechanisms underlying the subjects behavioral adaptation properties during the task, we simulated a series of different possible computational models and compared the ability of these models to fit individual subjects behavior on a trial-by-trial basis. All models are built on the assumption that subjects' behaviors rely on either reinforcement learning (RL) mechanisms, working memory (WM) mechanisms, or a contribution of both. In the latter case, we tested different models with different computational principles for the coordination of RL and WM.

In order to approximate the behavior, we use the discrete-time stochastic control of Markov Decision processes. We define the set of states *s* ∈ {*Blue, Red, Green*} for all possible color stimuli, the set of actions *a* ∈ {*Thumb, Index, Middle, Ring, Little*} for all the possible motor responses, and the set of possible outcomes *r* ∈ {0, 1}. At each time step, the agent observes a given state *s*_*t*_ and computes the probability of action *p*(*a*_*t*_|*s*_*t*_) from which an action is sampled. Then, the generative model is updated according to the outcome *r*_*t*_.

#### 2.2.1. Habitual strategy

We choose to model habitual behavior with a *Q*-*Learning* (Watkins and Dayan, 1992), one of the standard “model-free” RL algorithms. This algorithm is called “model-free” (Daw et al., 2005; Samejima and Doya, 2007) in the sense that it learns cached values associated to reactively selecting different actions in different states of the world without trying to acquire an internal model of the world which would have enabled to infer the consequences of performing a given action in a given state.

The aim of the algorithm is to compute the quality of each state-action couple, known as the optimal Q-function, by evaluating a temporal-difference error δ_*t*_ (Sutton and Barto, 1998). Given a current state *s*_*t*_, action *a*_*t*_, and reward *r*_*t*_, the TD error is equal to the difference between observed and predicted rewards and can be used to update the *Q*-value function of the couple (*s*_*t*_, *a*_*t*_) as in the following equation:

(1) $$Q(s_{t},a_{t})\leftarrow Q(s_{t},a_{t})+\alpha (r_{t}+\gamma \underset{a}{\max}Q(s_{t+1},a)-Q(s_{t},a_{t}))$$

Parameters α and γ are the learning rate and the discount factor, respectively. α controls the speed of convergence of the algorithm, whereas the discount factor γ determines the importance of future rewards.

Eventually, a probabilistic choice is made through a soft-max activation function:

(2) $$p(a|s_{t})=\frac{\exp\beta Q(s_{t},a)}{\sum_{a}\exp\beta Q(s_{t},a)}$$

This equation converts *Q*-values into action probabilities, and an inverse temperature parameter β controls the trade-off between exploitation and exploration. To summarize, two properties are inherent to this strategy: slow learning and fast decisions. Fast in the sense that making a decision only requires to compare a set of values associated to competing actions in a given state, without searching in working-memory or inferring long-term consequences of actions. This slow learning property directly affects the accuracy of the action choice.

#### 2.2.2. Deliberative strategy

We propose a Bayesian Working Memory (BWM) model for the goal-directed system. To summarize the main features of the BWM model: it stores a limited number of explicit descriptions of past trials. At the beginning of each block, the BWM is initialized as an empty list and each element is added in a chronological order. An element *t*_*i*_ in memory contains the information about one previous trial under the form of three probability mass functions: *p*(*s*|*t*_*i*_) is the probability of having observed a certain state, *p*(*a*|*s, t*_*i*_) is the probability of having performed a certain action given a state, and *p*(*r*|*a, s, t*_*i*_) is the probability of having observed an outcome given a state and an action. The probability of having observed a transition is not represented since the transition between stimuli is randomized. A parameter *N* controls the maximum number of elements maintained and the oldest elements are removed when the number of elements is larger than this capacity *N*. One memory item is added after each trial with the following rules:

(3) $$p(s|t_{1})\;=\left\{\begin{array}{ll} 1 & \text{if s is last state}\\ 0 & \text{otherwise} \end{array}\right\}$$

(4) $$p(a|s,t_{1})\;=\left\{\begin{array}{ll} 1 & \text{if a is last action}\\ 0 & \text{otherwise} \end{array}\right\}$$

(5) $$p(r=1|a,s,t_{1})\;=\left\{\begin{array}{ll} 1 & \text{if }r_{t}>0\\ 0 & \text{otherwise} \end{array}\right\}$$

After each trial, the memory list is updated by convolving a uniform distribution U to each items *p*(*s*|*t*_*i*_), *p*(*a*|*s, t*_*i*_), *p*(*r*|*a, s, t*_*i*_) to account for memory decay:

(6) $$p(..|..,t_{i})=(1-\epsilon )\;p(..|..,t_{i})+\epsilon U$$

The older a particular item has been stored in memory, the flatter becomes its probability mass functions, i.e., the higher the loss of information about this item. The noise quantity ϵ controls the degradation of the working memory contents.

At the decision step of each trial, the probability of action *p*(*a*|*s, t*_0 → *i*_) is computed iteratively using Bayes rule. The term *t*_0 → *i*_ represents the number of memory items processed and can change from trial to trial. To compute the probability of action, the first step is to compute a joint probability mass function:

(7) $$p(s,a,r|t_{0\to i})=p(s,a,r|t_{0\to i-1})\;+\;p(s|t_{i})p(a|s,t_{i})p(r|a,s,t_{i})$$

We make the hypothesis of independence between successive memory items, allowing them to be summed. The process of decision relative to the rules of the task starts with:

(8) $$p(a,r|s_{t},t_{0\to i})=\frac{p(s,a,r|t_{0\to i})}{p(s_{t})}$$

A state *s*_*t*_ is presented to the agent with certainty. Therefore, *p*(*s*_*t*_) = 1 which allows to compute *p*(*a, r*|*s, t*_0 → *i*_). Using Bayes rule, this probability is then reduced to:

(9) $$p(a|r,s_{t},t_{0\to i})=\frac{p(a,r|s_{t},t_{0\to i})}{\sum_{a}p(a,r|s_{t},t_{1\to i})}$$

In this task, there are only two possible outcomes *r* ∈ {0, 1}, and according to the rules of the task, only one action leads to a positive reward and actions associated with negative reward must be avoided. In the beginning of the task, only negative rewards *r* = 0 have been received and untried fingers must be favored. On the contrary, when the only possible action leading to a positive reward has been received, the probability for this action in next trials must be maximal. This reasoning is summarized in the next equation:

(10) $$Q(s_{t},a)=\frac{p(a|r=1,s_{t},t_{0\to i})}{p(a|r=0,s_{t},t_{0\to i})}$$

Contrary to the model-free system, we do not use a Soft-Max equation for computing *p*(*a*|*t*_0 → *i*_) but a simple normalization process is used, which allows to avoid an additional temperature parameter.

The index 0 → *i* represents the number of elements used for computing *p*(*s, a, r*) (if *t*_0_, no memory items are processed and the action is sampled from a uniform distribution). In this decision process, only a subset of the available information can be pertinent for the action choice. Stimuli are independent and not all elements should be processed. If the right action was performed on the previous trial, then the decision can rely only on the first element in the working memory (encoding the previous trial) and elements about wrong actions do not need to be used. On the contrary, all memory elements about a certain stimulus need to be processed when the agent is still searching for the correct answer. We resolved this issue by measuring the Shannon information entropy on action probabilities:

(11) $$H=-\sum_{a}(p(a|t_{0\to i})\times \log_{2}p(a|t_{0\to i}))$$

Thus, action selection is made when the level of entropy *H* is lower than a given threshold θ. To understand the suitability of entropy in this task, we can consider *p*(*a*|*r* = 1, *s*_*t*_) and *p*(*a*|*r* = 0, *s*_*t*_), which are two possible memory items of, respectively, positive and negative rewards that occurred when an action *j* has been performed on a previous trial. They are symmetrical since *p*(*a* = *j*|*r* = 1, *s*_*t*_) = *p*(*a* = *j*|*r* = 0, *s*_*t*_) (i.e., in two parallel worlds, the agent recalls that action *j* is positive/negative with the same probability). However, division in Equation (10) is not commutative and one can observe that $Q{\left(s_{t},a=j\right)}^{r=1}>Q{\left(s_{t},a=j\right)}^{r=0}$.

**Figure 2:**
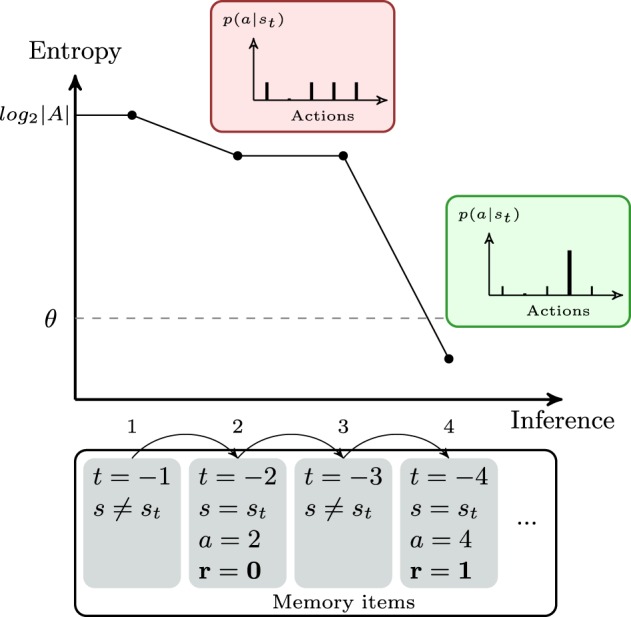
**Theoretical example of the evolution of the entropy during the decision process of the bayesian working memory model**. The agent is observing the current stimulus *s*_*t*_. In the bottom row, the set of memory items represents past trials in the chronological order. The first item (i.e., the previous trial) represents a different stimulus. Thus, no information is gained, the probability of action is uniform and the entropy *H*[*p*(*a*|*s*_*t*_)] is equal to the maximum entropy *log*_2_|*A*|. The second item is a negative recall (*r* = 0). Processing this item modifies the probability of action by suppressing the second action (see red box) and entropy decreases by only a small amount. The fourth trial is a positive recall (*r* = 1). The entropy is largely decreased by suppressing all the possible remaining actions(see green box). The threshold θ is crossed and the agent considers that he has enough information to stop searching in memory and to make a decision.

When normalizing *Q*-values, *p*(*a*)^*r* = 0^ is close to a uniform probability distribution minus one action—retrieving such an information from memory, thus, does not reduce much the entropy—while *p*(*a*)^*r* = 1^ is close to a Dirac function—which, thus, drastically reduce entropy when retrieved from memory. As trivial as it sounds, the gain of information is crucial because $H(p{\left(a\right)}^{r=1})\ll H(p{\left(a\right)}^{r=0})$. An illustration of the gain of information depending on the memory items processed is given in Figure 2. In this figure, the entropy *H* is plotted against the number of inferences when a particular set of memory items is processed. This property of our model is specific to the task since only one action leads to a positive reward for a given stimulus. More precisely, this rule of entropy minimization triggering decision is in mirror of the rules of the task. However, note that this does not prevent our model from generalizing to more statistical reward schedules. Of course, the parameter θ needs to be carefully estimated—it will be optimized for each subject—since it can force a decision to be made without certainty. In some cases, no information gain can occur after the evaluation of elements from the working memory, and decision is made as a consequence of absence of further items.

#### 2.2.3. Simulated reaction times

In the introduction of the instrumental learning task, we emphasized the importance of the evolution of reaction times and their potential relation with the hypothesis of dual-learning strategies. When designing the Bayesian Working Memory model, we naturally drew a parallel between the observed reaction times (supposed to reflect the subjects cognitive load) and the number of memory items processed that dynamically change from trial to trial depending on the gain of information.

In fact, the concept of evidence accumulation has already been explored in various race models and can account for a large variety of temporal observations (Reddi and Carpenter, 2000; Carpenter et al., 2009). Another pertinent work is the tentative of Norwich to unify laws of perception that predicts reaction time in a stimulus detection task (Norwich, 2003). He proposed that “as adaptation proceed, entropy (potential information) falls, and information is gained.” From this statement, a general descriptive model of reaction times (RT) for stimulus detection is derived. Very simply, the minimum quantity of information necessary to react can be quantified as a difference in entropy △*H* = *H*(*I, t*_0_)−*H*(*I, t*_*r*_) with *I* the stimulus intensity and *t*_*r*_−*t*_0_ the reaction time. To reduce entropy, the sensory receptors must be sampled *n* times in order to gain information and this sampling rate determines *t*_*r*_.

Following these ideas, we propose that the *simulated* reaction times sRT on the model are dependent of the logarithm of the number of processed items *i* plus the entropy computed from the final probability of action:

(12) $$sRT(trial)={\left(log_{2}(i+1)\right)}^{\sigma }+H(p(a|s_{t}))$$

σ is a free parameter controlling the proportion of the logarithm to the entropy in sRT. In the case of habitual strategy, *log*_2_(*i* + 1) is null and vRT is equal to the entropy computed over the probability of actions. In fact, *H*(*p*(*a*|*s*_*t*_)) will slowly decrease with the progress of habituation. We postulate that this variable can account for the overall habituation toward the structure of the task.

#### 2.2.4. Models of strategy coordination

**Figure 3:**
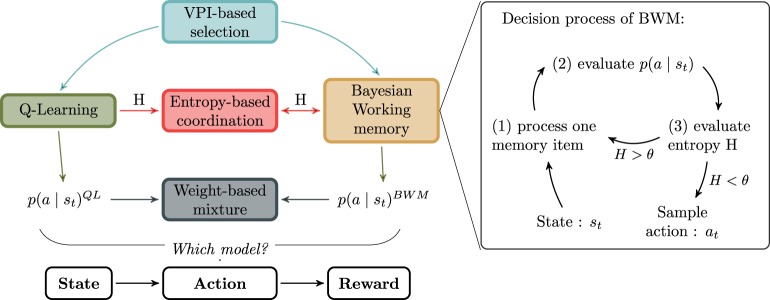
**Relationship between models**. The goal-directed and habitual strategy are respectively the Bayesian Working Memory (BWM) and the Q-Learning algorithm (QL). Between BWM and QL, the different models for the strategies interaction are respectively VPI-based selection, Entropy-based coordination or Weight-based mixture. In the right box, the decision process of BWM is decomposed into three stages. The purpose of the cycle is to reduce iteratively the entropy *H* computed over action probabilities.

So far, we have described a classical Q-Learning algorithm as habitual system and a new model of Bayesian Working Memory as goal-directed system. The two above-listed single-system models were meant to test the hypothesis that neither a Q-Learning nor a WM strategy alone can fully explain human behavioral adaptation performance in this task. In the following parts, potential models for strategy coordination are discussed. The summary of their relationship is presented in Figure 3 along with a sketch of the BWM process.

#### 2.2.5. VPI-based selection

The first interaction model is a process of selection directly adapted from Keramati et al. (2011). In this study, the effect of outcome-sensitivity depending on the duration of training is explained with a trade-off between speed and accuracy. The Value of Perfect Information (i.e., VPI-based selection) is proportional to the measure of uncertainty computed over the *Q*-values (see Mathematical Methods) in the habitual system, which decreases with training: with more uncertainty in the habitual system's action value distribution, the more information can be gained through searches in working-memory. For each *Q*(*s*_*t*_, *a*), the VPI is evaluated and compared with an exponential moving average reward rate $\bar{R}\left(s_{t+1}\right)\leftarrow \left(1-\sigma_{r}\right)\bar{R}\left(s_{t}\right)+\sigma_{r}r_{t}$. This variable specifies a cost for a high-level cognition: if reward rate is high and too much time is given to the goal-directed system, then the agent gains less rewards than if a fast decision is made. However, if uncertainty in the habitual system is too high, then it is still worthwhile losing time using the goal-directed system in order to make an appropriate decision. Finally, the following rules determine which among the two systems should make the decision on the next action to be performed by the agent. If $VPI\left(Q\left(s_{t},a\right)\right)>\bar{R}\left(s_{t}\right)$, then the best strategy for the agent is to use the BWM, which provides accurate *Q*-values. If $VPI\left(Q\left(s_{t},a\right)\right)<\bar{R}\left(s_{t}\right)$, then the number of positive rewards obtained for a given unit of time will increase if a fast decision is made from the QL with low uncertainty.

#### 2.2.6. Weight-based mixture

The second interaction model that we tested is the weight-based mixture model derived from Collins and Frank (2012). In this study, the interaction is specifically between a working memory model and QL. Despite the differences between their working memory model and BWM—i.e., their model do not use an adaptive number of search steps performed in working-memory, but rather used a fixed number of state, which is a parameter optimized for each subject—we integrated their main concepts: the decision results from a weighted sum of the output of each system, where the weights depends on the posterior likelihood of the systems:

(13) $$p(a|s_{t})=(1-w(t,s_{t}))p{\left(a|s_{t}\right)}^{QL}+w(t,s_{t})p{\left(a|s_{t}\right)}^{BWM}$$

The process of *w*(*t, s*_*t*_) evaluation, which determines the relative reliability of BWM over QL, is detailed in the supplementary section. Similar to VPI-based selection, here systems are separated and provide action probability distributions independently.

#### 2.2.7. Entropy-based coordination

In addition to these two models adapted from previous studies, we propose a third interaction model called entropy-based coordination, which explores the perspective of close interaction between strategies. The first point is to differentiate between the two measures of entropy associated with each individual strategy. *H*^*QL*^ is the entropy of information calculated upon the probability of action from the Q-Learning. This value decreases after each trial as the learning process progressively increases the difference between the value of the best action and other action values. It provides an information about the progression in the learning task. In contrast, *H*^*BWM*^ is evaluated within the working memory decision process. At the beginning of each trial, *H*^*BWM*^ is equal to the maximum value of information entropy $H^{max}=\log_{2}\left(\left|Action\right|\right)$. As elements in working memory are processed, *H*^*BWM*^ will decrease toward lower values (as illustrated in Figure 2). To summarize, *H*^*QL*^ evolves between trials, whereas *H*^*BWM*^ evolves within a trial.

The second point is the interplay between the strategies. Within working memory, we propose to replace the deterministic choice between deciding and retrieving memory items (threshold θ) with a binary probabilistic choice. Instead of comparing *H*^*BWM*^ with θ, one sub-action from the set {*deciding, retrieving*} is sampled with the probabilities $p\left(deciding|t_{0\to i},H^{BWM},H^{QL}\right)$ and $p\left(retrieving|t_{0\to i},H^{BWM},H^{QL}\right)=1-p\left(deciding|t_{0\to i},H^{BWM},H^{QL}\right)$. To sample one of the two possible sub-actions {*deciding, retrieving*} after each memory item process, these probabilities are computed with the following logistic equation:

(14) $$\begin{array}{l} p(Deciding|t_{0\to i},H^{BWM},H^{QL})\\ \;=\frac{1}{1+\lambda_{1}(n-i)\exp^{-\lambda_{2}(2H^{max}-H_{0\to i}^{BWM}-H^{QL})}} \end{array}$$

with *n* ≤ *N*, the number of elements stored in working memory at a given trial, *i* the number of items processed, and λ_*i*_, the decision gain. If the information about past trials is contained in working memory (i.e., *n* increases), it must be processed until *H*^*BWM*^ is low enough. Therefore, the increase of *n* favors the sub-action *retrieving* over the sub-action *deciding*. The variable *n* is indispensable in a goal-directed process since we want the agent to exploit as much available information as possible in order to choose the most accurate action. Nevertheless, the difference *n* − *i* behaves as a dynamical cost inside a trial that increases *p*(*deciding*) as memory items are processed. Indeed, decision must be made within a given time range. The probability *p*(*deciding*) is computed every time a memory item is recalled and enables to choose between searching for more information with probability 1 − *p*(*Deciding*) or engaging the decision process to sample the action. If the decision process is engaged, *Q*-values from each strategy (QL and BWM) are simply summed as in the following equation:

(15) $$Q(s_{t},a)=Q{\left(s_{t},a\right)}_{0\to i}^{BWM}+Q{\left(s_{t},a\right)}^{QL}$$

At last, action probabilities are computed within a softmax function (see Equation 2) with an inverse temperature β_*final*_ different from the temperature β used to normalize *Q*-values from the Q-Learning system. Except for the usual trade-off between exploitation and exploration, the softmax equation is important because of the translational symmetry that we can take advantage of. In fact, the final *Q*-values are always the sum of both systems. In the beginning of the task, the working memory can have extracted a lot of information inside the *Q*-values. And within the softmax function, the probabilities of actions are not disturbed by adding uniform *Q*-values from an ignorant Q-Learning. To understand this aspect, and in general the model, it is meaningful to illustrate three phases of the instrumental task.

1. In the first trial, working memory is an empty list, QL and BWM provide uniform *Q*-values and *H*^*QL*^ = *H*^*BWM*^ = *H*^*max*^. Therefore, *p*(*deciding*) = 1 and the decision is necessarily made. In fact, the agent starts the task without any clues.
2. In the acquisition phase, *H*^*QL*^ is close to *H*^*max*^ since Q-Learning is a slow-learning algorithm. After a few trials with negative outcomes, the number *n* of memory items increases in the BWM system. By combining those factors, *p*(*deciding*) is low at the onset of a trial and the process of retrieving in the BWM will be favored against a stimulus-based response from the QL system.
3. The consolidation phase starts when correct actions have been found. In such a case, *H*^*QL*^ is still gradually decreasing while *H*^*BWM*^ decreases within a few retrieval. Indeed, memory items start representing correct trials and the asymmetry of $Q{\left(s_{t},a\right)}^{BWM}$ between correct actions and incorrect actions is directly influencing *p*(*deciding*) (the entropy falls down instantly only when remembering correct actions). Nevertheless, memory items about correct trials are not the only influence on fast memory processing. The process of engramming action values within Q-Learning algorithm decreases *H*^*QL*^ toward zero. Consequently, *p*(*Decision*) is higher at the onset of a trial and decision is made more and more quickly during late trials of the task.

To summarize, the Entropy-based coordination balances the mechanism of BWM based on the uncertainty of the goal-directed and habitual strategies.

### 2.3. Methods for model comparisons

So far, we have presented five models (Q-Learning: QL only, Bayesian working memory: BWM only, Entropy-based coordination of QL and BWM, VPI-based selection between QL and BWM and Weight-based mixture of QL and BWM) that can choose actions and predict sRT following Equation (12). The best generative model is defined by his capacity to replicate subjects' trial-by-trial of action choices and reaction times. Since we have two objectives to fulfill through optimization of model parameters, this problem of model fitting is transposed into a multi-objective optimization framework that we choose to solve using the SFERES tool (Mouret and Doncieux, 2010). Using the standard evolutionary algorithm NSGA-2, an individual is defined as a vector of parameters θ_*model*_ for one model. Table 1 summarizes each model's free parameters The evolutionary algorithm consists in starting with a group of individuals with randomly initialized parameters constituting the first generation, and then iteratively selecting the best individuals generation after generation, in order to convergence on a set of parameter sets which best fit subjects' choices and reaction times. The NSGA-2 algorithm also includes in its fitness function a measure of diversity within the population, which has been shown to enable better convergence on the optimum for models with large number of free parameters (Mouret and Doncieux, 2010).

**Table 1:** **Parameters from single-learning strategies are also present in dual-learning strategies excepts for θ, which disappears in entropy-based coordination and α which disappears in VPI-based selection**.

**Model**	**Symbol**	**Range**	**Description**
Q-L only	α	0 < α < 1	Learning rate
	β	0 < β < 100	Softmax temperature
BWM only	*N*	1 < *N* < 10	Memory size
	θ	0 < θ < *log*|*A*|	Fixed entropy threshold
	ϵ	0 < ϵ < 0.1	Memory items decay
VPI-based selection	η	0.00001 < η < 0.001	Covariance initialization
	σ_*r*_	0 < σ_*r*_ < 1	Reward rate update
Weight-based mixture	*w*_0_	0 < *w*_0_ < 1	Initial weight
Entropy-based coordination	λ_1_, λ_2_	0.00001 < λ_*i*_ < 1000	Sigmoide parameters
	β_*final*_	0 < β_*final*_ < 100	Softmax temperature
	σ	0 < σ < 20	Simulated RT

At each generation, the best individuals are selected by generating the corresponding parameterized model and evaluating its capacity to maximize three fitness functions. Briefly, the first fitness function is the maximum likelihood that the model chooses the same action as the subjects (Daw et al., 2011). The second fitness function is a negative least-square error between mean RTs and sRTs averaged over representative steps as shown in Figure 1B. The third fitness function is a measure of diversity (i.e., distance) between the current parameter set and other parameter sets found in the population. A population of parameters is optimized independently for each subject and each model. After optimization, the evolutionary algorithm proposes a set of solutions *A* (i.e., parameters) that needs to maximize objective functions *f*_*i*_:*A* → ℝ, *i* = 1, …, *n* (in our case, *n* = 2). We note *f* = (*f*_1_, …, *f*_*n*_) the vector of objective functions. Given two solutions *a, b* ∈ *A*, the solution *a* dominates *b* if *f*_*i*_(*a*) ≥ *f*_*i*_(*b*), *i* = 1, …, *n* and there exists *i* such that *f*_*i*_(*a*) > *f*_*i*_(*b*). In other words, we keep only the solutions that are strictly better for, at least, one objective and this set of solutions constitutes the Pareto front (Deb et al., 2000). Thus, multiple solutions can exist and some compromises must be realized. To determine the best model for each subject, the first step is to combine the pareto front of the different putative models. The population of best individuals from each model and subject is mixed to select a new population of best individuals for each subject. We refer to aggregation as the process of combining the numerical coordinates ${\left\{x_{1},x_{2}\right\}}^{pareto}$ of a solution into a single one to construct a relation of preferences between solutions. Here the aim to verify the quality of sets of solutions by selecting only one solution. A large set of aggregation functions exists in the corresponding literature (Emrouznejad and Marra, 2014) but the aim of the work is not to compare nor study them. The aggregation functions deal with the compromise of losing a quality of fit in one dimension while gaining on the other. We tested three classical aggregation functions (namely Chebyshev, OWA, and Distance aggregation functions) and they generate probabilities of correct responses that were equivalent. In fact, most of the sets of solutions selected by aggregation functions overlap. We chose to focus on the results of one that we termed as Chebyshev aggregation function. The mathematical details of this function is provided in the mathematical methods section at the end of the paper.

We performed two versions of this optimization process: one on choice only (deactivating the second fitness function on reaction times) and one on both choice and reactions times (keeping all fitness functions active).

## 3. Results

### 3.1. Fitting results for action choices only

In a first part, we consider the fitting results for action choices only. The optimization process is made for each subject and each model, and returns, amongst others solutions, the solution $\theta_{model}^{max}$, which maximizes the likelihood function $\hat{L}=\sum P\left(choice|model,\theta_{model}\right)$.

**Figure 4:**
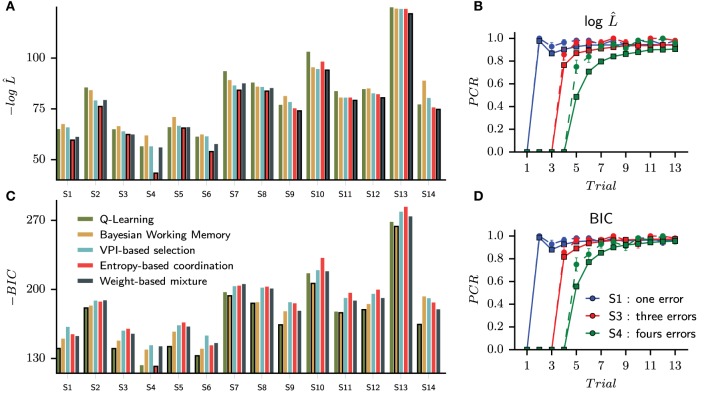
**Choice only optimization results and the probability of correct responses generated from the set of best models selected with a maximum likelihood criterion (A,B) and a Bayesian Information Criterion (C,D)**. **(A)** Without correction of the likelihood function, the set of best models is composed of eight Entropy-based coordinations and six weight-based mixtures. The bar of the best model is circled with a black line for each subject. **(C)** Applying the BIC, nine Q-Learning models, four Bayesian Working Memory models and one entropy-based coordination model are selected as best models. During free simulation of the latter optimized models, we can observe that the quality of the fit is slightly reduced for the raw likelihood **(B)** compared to the BIC **(D)**.

In this first straightforward approach, we assign to each subject the best model by comparing raw likelihoods. The goal is to see whether we can reproduce with our data Collins and Frank's observation (Collins and Frank, 2012) that the BIC criterion over penalizes the complexity of dual-system models, whereas the latter's raw likelihoods are better than single-systems' ones. We will next show model comparisons including a penalty term for the number of free parameters with the BIC criterion. We found that the entropy-based coordination model is the best model for eight subjects, whereas the Weight-based mixture best captures six subjects (Figure 4A).

To verify the ability of each winning model to replicate the behavioral results, we then tested each optimally parameterized model: each differently parameterized model is making its own choices different from those of the subject (as if each model was freely performing the task like the subjects did). Similar to Figure 1, we computed the probability of correct responses (PCR) for each stimulus averaged over all fourteen specifically parameterized models. The performance of the models is shown in Figure 4B with the corresponding human performances in the background.

While the probability of correct responses is mostly indistinguishable for S1 and S3 (respectively, the blue and red curves), we observed a large difference for S4 (the green curve). To compare subjects and simulated learning curves, we performed a Pearson χ_2_ test for each stimulus and each trial upon percentages of correct responses. We found nine significantly different trials between subjects and models performances. As it can be observed in Figure 4B, six discordant trials are for S4: trials 5, 6, 7, 8, 10, and 12. The largest difference is observed for the fifth presentation of S4 (Pearson χ_2_ test, 1 df, *t* = 15.52, *P* < 0.001). The simulated models are making more repetive errors than the subjects when searching for the correct answer and thus do not reach the average performances of the subjects during the consolidation phase.

Usually, the process of model selection is made by including a penalty term for model complexity (Daw, 2011; Khamassi et al., 2015). The most widely used is the Bayesian Information Criterion (Schwarz, 1978) which is an asymptotic approximation of a Bayesian posterior probability. The penaly term is computed according to the number of free parameters of each model. In our study, the simplest model is the Q-Learning (three free parameters) and the most complex model is the Entropy-based coordination (seven free parameters); see Table 1. When applying the BIC criterion, we find that the results change drastically, now favoring simpler models. The Q-Learning is assigned to nine subjects and the Bayesian Working Memory to four subjects. Only the Entropy-based coordination model for subject three survives the penalization process (Figure 4C) Thus, as in a previous study testing the relative contribution of RL and working memory in human subjects performances (Collins and Frank, 2012), the use of a penalty term for complexity favors single-learning systems over dual-learning systems. The results from testing the set of best models selected with BIC is shown in Figure 4D. From the statistical test, we found that trial 5 is significantly different (Pearson χ_2_ test, 1 df, *t* = 8.44, *p* < 0.01).

In the next analysis phase, we will see that fitting models on both choices and reaction times (which is one of the main novelties of this work) drastically reduce the ability of single-system models to fit subjects' behavioral data compared with dual-systems.

### 3.2. The pareto fronts of the fit on choices and reaction times

**Figure 5:**
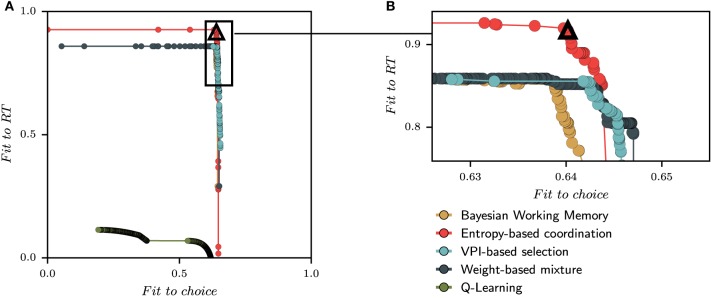
**Set of best solutions for each model for subject 7 obtained with the multi-objective parameter optimization on choices and reaction times**. One point represents one parametrization for one model and one subject evaluated on the *fit to RT* and the *fit to choices*. **(A)** At the end of the optimization process, the pareto front for one subject is constructed by conserving only solutions, regardless of the model, that can not be improved in one dimension without being worsened in the other. The best solutions are located in the top-right corner of each subfigure. **(B)** The top right corner of pareto fronts is enlarged to show the relative position of each model. The black triangle is the position of the selected solution which is the best compromise between the quality of fit to choice and the quality of fit to RT (see Section 5). For this subject, the best solution is given by the Entropy-based coordination model since it increases largely the quality of fit to RT compared to other models. This model selection process is made for each subjects.

We next performed a new parameter optimization for each model, this time with the multi-objective of fitting both choices and reaction times. The Pareto fronts of all models for one subject (Subject #7) is shown in Figure 5. Each point on the Pareto front represents one parametrization for one model and one subject, which “dominates” (in the Pareto sense) all suboptimal parameter sets and which is itself not dominated by any other parameter set. The solutions for the same model are in the same color and connected through one line. Maximizing the fit on both choices and reaction times can graphically be interpreted as the population of solutions that forms the Pareto fronts to move toward the optimal solution located in the upper right corner of the Figure 5A.

From the size and positions of the Pareto fronts, we can already observe a diversity of solutions. For instance, the Q-Learning model shows a very bad quality of fit to the reaction times compared to other models. In Figure 5B, a zoom is made to the corner of the Pareto fronts. Starting from the best fit to choice in the x-axis, the three dual-learning systems are mostly equivalent. As one climb the Pareto fronts, one progressively looses the quality of fit to choice while improving the quality of fit to RT. At some point, a dissociation between models is observed since the entropy-based coordination is climbing higher than the two other models. For this subject, the entropy-based coordination gives a better fit to RT compared to other models. Therefore, selecting a solution within the Pareto front brings the question of the acceptable amount of fit to choice we can lose to obtain a better fit of RT.

In order to perform tests, we select one solution for each subjects from this population of solutions containing several models. In the example given in Figure 5A, the pool of possible solutions only contains parameterizations of dual-learning systems (Entropy-based coordination, Weight-based mixture, and VPI-based selection) and excludes single-learning systems (Q-Learning and Bayesian Working Memory). In other words, the front of single learning strategies is fully dominated by dual-learning strategies, and the selection of dominating fronts is only composed of solutions from dual-learning strategies.

The process of selecting the best solution within a Pareto front is a complex issue in the field of multiple-criteria decision-making (Zitzler and Thiele, 1999). We follow previous proposals to solve this issue by using the Chebyshev aggregation function, the latter being the process of combining the numerical coordinates ${\left\{x_{1},x_{2}\right\}}^{pareto}$ of a solution into a single one to construct a relation of preferences between solutions (Wierzbicki, 1986). In Figure 5, the position of the solution selected with the Chebyshev aggregation function for subject 7 is illustrated with a black triangle.

This aggregation process gave a set of best models for all subjects composed of nine Entropy-based coordinations, three weight-based mixtures, and two VPI-based selections. While different from the first set of models is selected in the action choices, the supremacy of dual-learning systems is clearly established.

To verify the robustness of our methods, we perform a leave-one-out method in order to validate that each subject is assigned the best model. One bloc out of four is systematically removed from the training set. The optimization is made upon choices and reaction times for all subjects with only three blocs. The same aggregation function is applied in order to compare the best model. Results are shown in Table 2. The second to fifth columns are the results with one bloc left outside of the optimization. The last column are the original results. Bold cells indicate discordand results. Only three subjects (1, 7, and 9) give, in a majority of test, a different result. For six subjects out of 14, the results are the same (3, 4, 10, 11, 13, and 14). In addition, the best model is the same in three test for three subjects (2, 5, and 6). Overall, the percentage of errors is 30% (17/56). Besides, the most striking observation is the supremacy of dual-strategy models that appears in all cases. The leave-one-out test asserts the hypothesis that a combination of strategy is required to explain the results.

**Table 2:** **Results from leave-one-out validation**.

**Subject**	**-Bloc 1**	**-Bloc 2**	**-Bloc 3**	**-Bloc 4**	**All blocs**
1	**E-Coord**	W-Mix	**E-Coord**	**E-Coord**	W-Mix
2	**VPI-select**	E-Coord	E-Coord	E-Coord	E-Coord
3	E-Coord	E-Coord	E-Coord	E-Coord	E-Coord
4	E-Coord	E-Coord	E-Coord	E-Coord	E-Coord
5	**W-Mix**	E-Coord	E-Coord	E-Coord	E-Coord
6	E-Coord	E-Coord	E-Coord	**W-Mix**	E-Coord
7	**E-Coord**	**VPI-select**	**VPI-select**	W-Mix	W-Mix
8	**W-Mix**	VPI-select	VPI-select	**E-Coord**	VPI-select
9	**VPI-select**	**VPI-select**	**VPI-select**	**VPI-select**	W-Mix
10	VPI-select	VPI-select	VPI-select	VPI-select	VPI-select
11	E-Coord	E-Coord	E-Coord	E-Coord	E-Coord
12	E-Coord	**W-Mix**	E-Coord	**W-Mix**	E-Coord
13	E-Coord	E-Coord	E-Coord	E-Coord	E-Coord
14	E-Coord	E-Coord	E-Coord	E-Coord	E-Coord

*Each bloc is removed systematically for optimization. Discordant models compared with optimization with all blocs are shown in bold. (Q-L, Q-Learning; BWM, Bayesian Working Memory; VPI-select, VPI-based selection; W-Mix, Weight-based mixture; E-Coord, Entropy-based coordination)*.

### 3.3. Comparing the fit of dual- and single- learning systems to choices and reaction times

In the following part, each parameterized model is tested with the same stimulus order than the related subject. In order to appreciate the quality of fit of the set of best models and to disentangle the contribution of each strategy, the Tchebytchev aggregation function is also applied to each individual strategy to select a set of solutions for the Q-Learning and a set of solutions for the Bayesian Working Memory. Concretely, we simulate an average behavior over choices and reactions times for the set of best Q-Learning parameterizations, the set of best Bayesian Working Memory parameterizations, and the set of best models.

**Figure 6:**
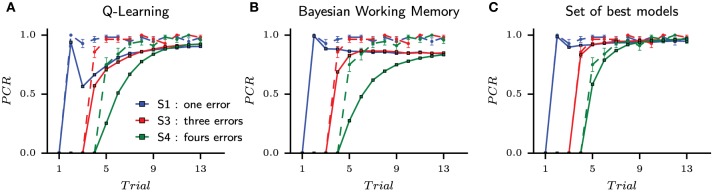
**The probability of correct responses (PCR) from the habitual strategy (Q-Learning), the goal-directed strategy (Bayesian Working Memory) and the best models according to the performed multi-objective optimization**. Each model is tested with the same block of stimuli than the corresponding subjects and the modeling results are superposed with subjects behavioral results (dashed lines). The blue, red and green curves (one per stimulus) represents the probability of correct response vs. the number of stimulus presentations computed on the sequence of outcomes (1 for correct and 0 for incorrect). **(A)** is the habitual strategy only. **(B)** is the goal-directed strategy only. **(C)** is the set of best models when mixing Pareto fronts as shown in Figure 5. In this case, the set of best models is composed of nine entropy-based coordinations, three weight-based mixtures and two VPI-based selections, i.e., only dual-learning strategies.

The behavioral learning curves from the simulation of fourteen parameterized models are shown in Figure 6 for single strategies (Figure 6A for QL and Figure 6B for BWM) and for the best models (Figure 6C). Each generated behavior is superposed with the human learning curves in dashed lines. For each test, the probability of correct responses (PCR) is computed over the sequence of binary outcomes for each stimulus as in Figures 1B, 4.

For the set of best models and like the best models previously selected with BIC on choices, only the fifth trial of S4 showed a significant difference in the probability of correct responses between the simulated and subjects' responses (Pearson χ_2_ test, *T* = 5.57, *p* < 0.05). In none of the other 44 cases, significant difference between models and subjects was found (Pearson χ_2_ test, *T* < 2.16, *p* > 0.14). Concerning the set of best Q-Learning models, 18 trials over 36 were significantly different (Pearson χ_2_ test, *T* > 4.04, *p* < 0.05). Most of these discordant trials were found in the beginning of the task (Figure 6A). For the set of best Bayesian Working Memory models, 22 trials were significantly different from subjects' choices (Pearson χ_2_ test, *T* > 3.87, *p* < 0.05) as shown in Figure 6B.

Concerning S1, only the BWM models and the best models manage to reproduce subjects' performances at the first correct response at the second trial. On average, the performance of the QL model was lower ($PCR_{blue,\;second\;trial}^{Q-L}=98.6\%$). At the second trial, the penalty upon the probability of the action chosen in the first trial is not large enough in these modes to prevent from being selected in the second trial. This is because the Q-Learning model is a slow learning algorithm. In the following trials, the performance of Q-Learning models falls to about 60% and then slowly increases back. For the Bayesian Working Memory models, the performance falls to 80% and remains constant for the following trials until the end of the block, unlike subjects' performances that continue to gradually increase until the end. Except for a slightly drop of performances at the first trial, the set of best models provides performances that are not statistically different from the subjects for stimulus S1.

For the remaining stimuli, the subjects' average performances are captured by the set of best models. The models' probabilities of correct response at the fifth trial for S3 are lower than that of the subjects. Since at this trial only one possible action remains (the four others having previously been associated to errors), this means that the dual-learning models are making slightly more repetitive errors than the subjects. For the Q-Learning model, the performances for S3 and S4 are below subjects' performances except for the last trials. Such an observation illustrates the slow convergence property of the habitual strategy. As stated above, this also contrasts with the goal-directed strategy whose performances for all stimuli converge to a steady probability of correct responses that does not evolve along trials.

**Figure 7:**
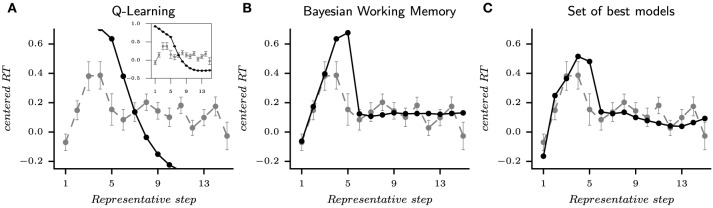
**Reaction times from the habitual strategy (Q-Learning), the goal-directed strategy (Bayesian Working Memory) and the set of best models according to the performed multi-objective optimization**. The distribution of RTs from a model or a subject are standardized according to the respective median and interquartile range. To represents the evolution of mean reaction times, the average is computed according to representative steps. The acquisition phase ranges from step 1 to 5 and represents the required mistakes for each stimulus and the first correct response. The consolidation phase starts at step 6 and starts after the correct response is given to the subject (or model). For each figure, the black line is the simulated reaction times and the gray lines is the human reaction times. **(A)** is the habitual strategy. The inserted figure shows the full curve. **(B)** is the goal-directed strategy. **(C)** is the set of best models when mixing Pareto fronts as shown in Figure 5. This behavior is extracted from the same simulation than the one shown in Figure 6.

The second behavioral measurement from the experiment is the reaction time (RT). Reaction time results are shown in Figure 7 extracted from the same generated behavior than Figure 6 (i.e., the same set of parameters for each model). In this figure, we applied two consecutive processes: rescaling and reordering in order to compare and appreciate the evolution of mean reaction times between model simulations and subjects' data. Rescaling is necessary since we compare a distribution of reaction times in seconds with a distribution of simulated reaction times in arbitrary units. We choose to standardize each distribution according to the respective median and interquartile range. The process of ordering is the same than in Figure 1B and applied to the simulated reaction times (sRTs) generated by the model. Once again, subjects and simulated behaviors are displayed on top of each other in Figure 7 (gray dashed line for RTs and black full line for sRTs).

For the generated behavior of the Q-Learning, we observe that the sRTs are only decreasing. In fact, the sRTs of Q-L are computed as the entropy of the final probability of actions (see Section 5). In other words, the habitual strategy is becoming faster in responding along learning, which differs from subjects' behaviors. This result recalls the previous observation that the QL algorithm's “fit to RT” as illustrated with the Pareto fronts of subject 7 (Figure 5) was not the best capture on reaction times behavior. The Bayesian Working Memory model provides a richer behavior since we observe an increase in reaction times from representative steps one to five followed by a decrease to an almost constant but slightly fluctuating value in the following steps. Despite the fact that this up-and-down evolution is also present in subjects' RTs, we can observe a discordance between the two distributions. With a Mann–Whitney *U*-test, we found seven significatively different representative steps (Mann–Whitney *U*-test, *p* < 0.05). At last, the evolution of subjects' RTs is replicated in a better way by the set of best models with six significantly different representative steps (Mann–Whitney *U*-test, *p* < 0.05). During the consolidation phase, sRTs gradually decrease due to the contribution of the Q-Learning model, more similar to the evolution of subjects' RTs (Figure 7C) Nevertheless, the large difference is still observed for the fifth step.

**Figure 8:**
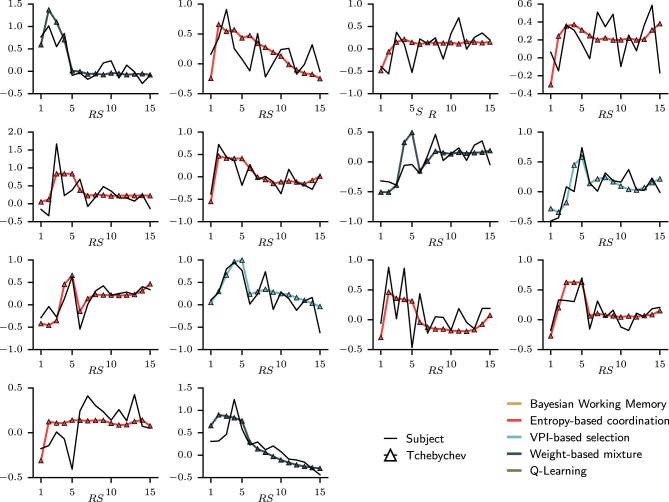
**Fit on individual subjects' reaction times displayed over representative steps**. In each sub-figure, the black full line is the mean reaction time for one subject averaged over representative steps. To compare with the simulated reaction time, each distribution of RTs is standardized according to the respective median and interquartile range. Over the subjects' RTs, the simulated RT is plotted with a color indicating which type of model was chosen. In this case, only dual-learning systems have been selected as best models to explain the evolution of mean reaction times.

The quality of the fit to RT on a subject-by-subject basis can be apprehended in Figure 8. For each subject, the evolution of mean RTs over representative steps is plotted against the simulated mean RTs. In others words, the global mean RTs shown in Figure 7 can be separated into individual's mean RTs as shown in Figure 8. Looking at the evolution of RTs for each subjects, we can observe the unstable and noisy measurements that constitute RTs, resulting in somewhat substantial inter-individual differences. Yet, the sRTs generated by the set of best models are not standardized variables that match only the average RTs over all subjects since optimization has been performed separately for each subject. It, thus, manages to follow the global evolution of individual RTs. The figure also shows that despite inter-individual differences, some of the main tendencies of the model—i.e., increase in RT during the first trial; progressive decrease during late trials—are observed in the majority of subjects.

### 3.4. Relative contribution in a dual learning systems

**Figure 9:**
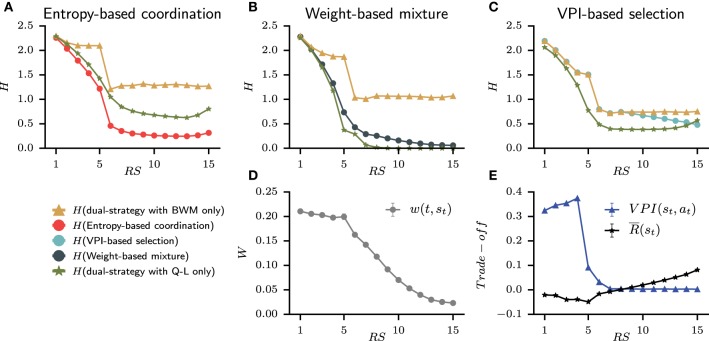
**Average contribution at each representative steps of the goal-directed and habitual strategy for each model from the set of best model**. **(A–C)** For each model, the contribution is evaluated by removing *Q*-values from one strategy and comparing the resulting entropy H of probability of action. The green triangle line is the final entropy from the dual-strategy with BWM only. The yellow star line is the final entropy from the dual-strategy with Q-L only. Also plotted with circles is the final entropy of the full model (i.e., BWM+Q-L). **(D)** The weight from the Weight-based mixture model that shows the reliability of BWM over QL. **(E)** The speed-accuracy trade-off of the VPI-based selection shows a switch of strategy around trial 8.

Since the set of best models is only composed of dual-learning systems, it allows us to explore the simulated relative contribution of the goal-directed and habitual strategies, which best accounts for each subject's behavior in this task. The results are shown in Figure 9 averaged over subjects with the same best models. For the weight-based mixture, the relative contribution is embodied into the weight *w*(*t, s*_*t*_) that evolves through trials. Moreover, the VPI-based selection with the speed-accuracy trade-off is straightforward. However, the nature of the entropy-based coordination makes contribution from each strategy more complex to evaluate. Therefore, we rely on the technique of “lesioning” one part of a cognitive system to observe the resulting activity that we measure through entropy. At each time step of behavior simulation, the entropy *H* is evaluated from the probability of action with both BWM and QL, and the probability of action without the contribution of *Q*-values from either BWM and QL. This lesion study is shown in Figures 9A–C for each model of dual-learning strategies.

The most striking observation is that only H(entropy-based coordination) is lower than H(entropy-based coordination with BWM only) and H(entropy-based coordination with Q-L only). In other words, the quantity of information contained in the probability of action when combining strategies is greater than one strategy alone. This is different from weight-based mixture and VPI-based selection for which H(dual-strategy) is bounded between the entropy of the lesioned models. Therefore, the entropy-based coordination is the only model to display emerging gain of information when combining the habitual and the goal-directed strategy. We also observed that the weight-based mixture shows a clear preference for the habitual strategy with a small weight that only decreases (i.e., thus favoring Q-L, Figure 9D). The weight decreases monotonically, meaning that the contribution of BWM decreases through time, whereas the contribution of QL increases. However, the weight is small, meaning that the contribution of QL is always higher than that of BWM. This is also observed in Figure 9B with H(Weight-based mixture) closer to H(weight-based mixture with Q-L only). Nevertheless, a contribution of BWM is necessary from Step 1 to 5 in order to solve the task. Finally, the VPI-based selection model displays a coherent behavior with the hypothesis that it incarnates, i.e., speed-accuracy trade off with the goal-directed strategy in the beginning of the task and the habitual strategy at the end of the task. The fact that the VPI is higher than the reward rate during the acquisition trials favors BWM [i.e., H(VPI-based selection) is equal to H(VPI-based selection with BWM only)]. At the fifth and sixth steps, the VPI falls down, which corresponds to the end of the acquisition phase. The agent have received all the correct answers and the VPI decreases abruptly. Still, the VPI is higher than the reward rate, and a switch of strategy is observed around trial 8 as the reward rate is higher than the VPI. In Figure 9C, H(VPI-based selection) slowly approaches H(VPI-based selection with Q-L only).

## 4. Discussion

In this study, we fitted the behavioral results from the instrumental learning task designed by Brovelli et al. (2008, 2011) to study the interactions of the goal-directed and habitual systems. We proposed a new model of Bayesian Working Memory as a goal-directed strategy. We also proposed entropy-based coordination as a new model of coupling between goal-directed and habitual strategies (Q-Learning algorithm). To compare with the corresponding literature, we adapted models of strategy interaction as Weight-based mixture from Collins and Frank (2012) and VPI-based selection from Keramati et al. (2011). To optimize free parameters of each best possible model (i.e., single learning strategies or dual-learning strategies), we used the multi-objective evolutionary algorithm NSGA-2 (Mouret and Doncieux, 2010) applied to each subject's behavior measured as choice and reaction time. In addition, we used a diversity function to ensure the convergence of the optimization process.

In the first step, we performed optimization upon choices only and the raw likelihood favored dual-learning strategies. Nevertheless, we found that applying a complexity penalty upon likelihood score (Schwarz, 1978) drastically changed the result of the optimization. The single learning strategies, with a fewer number of parameters, were assigned as best models to explain choices in this case.

In the second step, we constrained the evolutionary algorithm to minimize, along with the likelihood of the choices, the least-square error between subjects' average reaction times and models' simulated reaction times. Selecting solutions from subjects' Pareto fronts gave a set of only dual-learning strategies as best models to capture the full behavioral observations of the instrumental learning task.

The set of best dual-learning strategies is composed of nine entropy-based coordinations, three weight-based mixtures, and two VPI-based selections. Despite the fact that the entropy-based coordination is assigned to more subjects than the two other models, we have not found a clear supremacy of one dual-system model over the others. Each model of dual-learning strategy provides a particular mode of interaction that explains a matching subject. On the one hand, this accounts for part of inter-individual differences; on the other hand, this absence of supremacy raises a question about the possibility of a more general model to approximate each dual-system model depending on the subject. Through the literature, all proposed models can be reduced to three possible modes of interaction that we have compared (i.e., selection, coordination, and mixture of strategies). In Chavarriaga et al. (2005); Daw et al. (2005); Dollé et al. (2010); Keramati et al. (2011); Pezzulo et al. (2013); Renaudo et al. (2014), the mode of interaction is selection. In Wiering and van Hasselt (2008); Gläscher et al. (2010); Collins and Frank (2012); Lesaint et al. (2014), the mode of interaction is mixture based on weighting. Coordination as a model of interaction is harder to define since single learning strategies can be implicit. An example is provided in Dezfouli and Balleine (2012) where action sequences (i.e., habitual strategy) are a full part of the goal-directed strategy. To our knowledge, no studies have ever tried to systematically compare all the different approaches of coordination of strategies.

We demonstrated the limitations upon fitting only choices while not taking reaction times into account with our specific dataset. Such an issue may reveal itself as important in the future in other datasets from decision-making studies. As illustrated here, a diversity of solutions exists when trying to fit both choices and reaction times, i.e., when transposing the problem of finding the best parameters to a multi-objective framework. These observations might also call for a reinterpretation of some previous results if reaction times were to be included. In fact, a notable example of multi-objective optimization in a neuroscientific computational model is provided in Liénard and Girard (2014). The authors demonstrated the existence of a set of good solutions for a basal ganglia model that would satisfy the corresponding biological observations. The counterpart of the multi-objective optimization is the selection of one solution among a set of possible solutions. Besides, the quality of the compromises depends largely on the shape of the Pareto fronts. For instance, the compromises within a right-angled shape can easily be found in the corner. On the contrary, the compromises are tricky in a flat Pareto front. In fact, information about a model can be gained from the shape of a Pareto front (Doncieux et al., 2015). To overcome these limitations, one solution would be to confront each model with several decision-making tasks to build systematic comparisons.

For years, the study of reward-based learning has mostly been concentrated upon model-free learning with great successes (Schultz et al., 1997; O'Doherty et al., 2004). Yet, the influence of higher level cognitive systems in such instrumental learning paradigms has never been neglected but needs to be computationally explained (Miller and Cohen, 2001; Donoso et al., 2014). In this work, the combination of reinforcement learning and working memory proved to be more efficient to capture the behavioral observations from an instrumental learning task. In other words, neither model-free learning alone nor working memory alone are sufficient as shown in Collins and Frank (2012). Complex cognitive systems require the ground provided by reinforcement learning algorithms. This hypothesis is conforted in this work by the approximation of reaction times with a dual-learning model. The same kind of deterministic task is used in monkey studies (Quilodran et al., 2008). The focus there has been made on reinforcement learning but a contribution of working memory is not to be excluded (Khamassi et al., 2015). More work is required to investigate further whether humans and non-human primates rely on a similar combination of RL and WM processes in this type of task.

Most results about reinforcement learning come from simplified and idealized paradigms. In this study, the instrumental learning task is composed of only three states and five actions. The low dimensionality of this environment allows a simple model of Bayesian Working Memory to work properly. Yet, it is not the goal of the analysis proposed here to capture all the phenomenological observations associated with working memory (Baddeley, 1992). Besides, the proposed model of working memory embodies a process of decision that depends on the rules of the task (i.e., only one action is associated with a correct answer). While the proposed model of Bayesian Working Memory lacks generalization, the three processes of combination that we compared can be tested on more complex and realistic tasks. In the robotic world, the number of states and actions is definitely larger than in the computational neuroscience world. Studies have already tried to adapt dual-learning strategy (Caluwaerts et al., 2012; Jauffret et al., 2013; Renaudo et al., 2014, 2015) and some hypotheses appear to be inadequate. For instance, the assumption that the model-based system has perfect information for action values (Keramati et al., 2011) does not make sense in a rich, fast, and dynamic world. The number of states or transitions is too important and complex. A tree-search does not have enough time to compute the values of all the possibilities even in the beginning of a task. Further inter-disciplinary works and exchanges between computational neuroscience and robotics could be fruitful in helping understand how humans, animals, and robots can efficiently coordinate multiple learning and decision-making systems to display robust efficient adaptive behaviors.

## 5. Materials and methods

### 5.1. Ethics statement

Fourteen healthy subjects participated in the study (all were right handed and seven were females; average age 26 years old). All participants gave written informed consent according to established institutional guidelines and they received monetary compensation (45 euros) for their participation. The project has been approved by the Comité Consultatif de Protection des Personnes dans la Recherche Biomédicale de Marseille 1 (50).

### 5.2. VPI-based selection

Strategies are selected according to a trade-off between speed and accuracy, and BWM and QL are fully segregated. The *Q*-values are computed independently and chosen according to a comparison between a Value of Perfect Information (VPI) and a reward rate $\bar{R}$ (Keramati et al., 2011).

#### 5.2.1. Value of perfect information

When observing one particular state *s*_*t*_, *VPI*(*s*_*t*_, *a*) is evaluated from the Q-Learning algorithm according to Equation (16). The burden of this method is the requirement for *Q*-values to be represented with normal distributions $N\left(Q{\left(s_{t},a\right)}^{QL},\sigma^{2}\left(s_{t},a\right)\right)$. This issue is resolved by the use of Kalman Q-Learning algorithm as described in Geist et al. (2009). The learning is also based on reward prediction errors, and after convergence, the relative position of the mean of each normal distribution is equivalent to those of a simple Q-Learning algorithm. If the environment is stationary and the rewards values are constant, $\sigma^{2}\left(s_{t},a\right)$ will decrease, reflecting a low uncertainty and the confidence of the Kalman Q-Learning algorithm in predicting the correct reward. On the contrary, unpredictable environments will increase $\sigma^{2}\left(s_{t},a\right)$. This property and the relative position of each normal distribution is exploited in Equation (16) to compute VPI, where *a*_1_ and *a*_2_ are the best and the second best actions, respectively, at state *s*_*t*_.

(16) $$\begin{array}{l} VPI(s_{t},a)\\ =\{\begin{array}{l} (Q{\left(s_{t},a_{2}\right)}^{QL}-Q{\left(s_{t},a\right)}^{QL})P(Q{\left(s_{t},a\right)}^{QL}<Q{\left(s_{t},a_{2}\right)}^{QL})+\\ \frac{\sigma (s_{t},a)}{\sqrt{2\pi }}\exp^{-{\left(Q{\left(s_{t},a_{2}\right)}^{QL}-Q{\left(s_{t},a\right)}^{QL}\right)}^{2}/2\sigma^{2}(s_{t},a)}\text{if }a=a_{1}\\ (Q{\left(s_{t},a\right)}^{QL}-Q{\left(s_{t},a_{1}\right)}^{QL})P(Q{\left(s_{t},a\right)}^{QL}>Q{\left(s_{t},a_{1}\right)}^{QL})+\\ \frac{\sigma (s_{t},a)}{\sqrt{2\pi }}\exp^{-{\left(Q{\left(s_{t},a_{1}\right)}^{QL}-Q{\left(s_{t},a\right)}^{QL}\right)}^{2}/2\sigma^{2}(s_{t},a)}\text{if }a\neq a_{1} \end{array} \end{array}$$

If *a* = *a*_1_, then $P\left(Q{\left(s_{t},a_{1}\right)}^{QL}<Q{\left(s_{t},a_{2}\right)}^{QL}\right)$ is obtained from the cumulative distribution function of the normal law and measures the overlap between the best action and the second best action. Similarly, $Q{\left(s_{t},a_{2}\right)}^{QL}-Q{\left(s_{t},a_{1}\right)}^{QL}$ is the distance between the center of the two gaussian distribution.

#### 5.2.2. Speed/accuracy trade-off

The selection between strategies is made according to the following rules:

(17) $$Q(s_{t},a)=\{\begin{array}{ll} Q{\left(s_{t},a\right)}^{BWM} & \text{ if }VPI(s_{t},a)>\bar{R}(s_{t})\\ Q{\left(s_{t},a\right)}^{QL} & \text{ if }VPI(s_{t},a)<\bar{R}(s_{t}) \end{array}$$

In the first condition, the uncertainty *VPI* is larger than the reward rate $\hat{R}$, and the action must be evaluated accurately. In the inverted second condition, the reward rate is larger than the uncertainty and the action must be sampled rapidly. Once *Q*-values are selected from one of the strategies, then probabilities of action are computed within a Softmax function as in Equation (2).

#### 5.3. Weight-based mixture

In Collins and Frank (2012), a model mixing of working memory and reinforcement learning in the decision process has been proposed. At the time of reward, the first step is to compute the likelihood of each strategy $p{\left(r_{t}|s_{t},a_{t}\right)}^{strategy}$ with the following equation:

(18) $$p{\left(r_{t}|s_{t},a_{t}\right)}^{strategy}=\{\begin{array}{ll} p{\left(a_{t}|s_{t}\right)}^{strategy}\; & \text{if }r_{t}=1\\ 1-p{\left(a_{t}|s_{t}\right)}^{strategy}\; & \text{if }r_{t}=0 \end{array}$$

Then, *w*(*t, s*_*t*_) is updated according to:

(19) $$\begin{array}{l} w(t+1,s_{t})\\ =\frac{p{\left(r_{t}|s_{t},a_{t}\right)}^{BWM}w(t,s_{t})}{p{\left(r_{t}|s_{t},a_{t}\right)}^{BWM}w(t,s_{t})+p{\left(r_{t}|s_{t},a_{t}\right)}^{QL}(1-w(t,s_{t}))} \end{array}$$

Therefore, the weight *w*(*t, s*_*t*_) will move proportionally to the confidence in the goal-directed strategy. At the decision step, a softmax function is used only for the habitual strategy to compute probabilities of action $p{\left(a|s_{t}\right)}^{QL}$ with an inverse temperature β.

### 5.4. Multi-objective optimization

The optimization process maximizes three fitness functions: the quality of fit to choices, the quality of fit to reaction times, and the diversity within a population of parameters. This diversity ensures a better convergence of the algorithm by fully exploring a continuous space of parameters (Mouret and Doncieux, 2012).

#### 5.4.1. Fitness functions

To avoid any pittfall in our study, we conducted trial by trial analyses (Daw et al., 2011) for each subjects. The first fitness function is the likelihood $\hat{L}$ that the model chooses the same action as the subject:

(20) $$\hat{L}=\prod_{t}p(a=a_{t}^{subject}|s_{t})$$

The second fitness function evaluates the ability of the generative model to predict the evolution of mean RTs. In the first step, the distribution of RTs and sRTs are standardized using their respective median and interquartile range. In the second step, RTs and sRTs are averaged according to representative steps. Finally, the fitness function is a simple least square error that must be maximized:

(21) $$E_{RT}=-\sum_{steps}{\left(RT_{step}-vRT_{step}\right)}^{2}$$

#### 5.4.2. Aggregation functions

Aggregation refers to the process of combining numerical values *x*_1_, …, *x*_*m*_ into a single one *M*(*x*_1_, …, *x*_*m*_) so that the final results of the aggregation takes into account all the individual values. In our problem, a solution is in 2 dimensions and the aggregations functions provides ranking of solutions according to their respective positions within a Pareto fronts. To be able to compare solutions, each objective value must be scalarized in order to belong to the unit interval [0, 1]. To normalize, the upper and lower bounds are, respectively, the best and worst values for each fitness function.

One way to define a scalarizing function in multi-objective optimization problems is to measure the distance to a reference point *p* ∈ ℝ^*m*^. In the simplest aggregation function, the solutions are ranked according to their *distance* to a reference point *p* = 1. This point corresponds to the upper bound for each fitness function. The solution with the lowest euclidian distance to the reference point is selected.

This method can be refined using a *Chebyshev* distance. The quality of the solution is defined as the distance to the target with the use of the infinite norm. A weighting vector $\lambda \in {\mathbb{R}}_{+}^{m}$ is introduced to bias the ranking if some criteria are more important than others. In this case, the reference point *p* is the *ideal* point α ∈ ℝ^*m*^ defined as α_*i*_ = *sup*_*x*ϵ𝕏_*x*_*i*_. One can note that the ideal point is different for each pareto fronts. Inversely, the Nadir (worst combination of criterion scores) is defined as β_*i*_ = *inf*_*x*ϵ𝕏_*x*_*i*_. Finally, the aggregation function is defined as:

(22) $$t(x)=\underset{i\in M}{\max}\lambda_{i}\frac{\alpha_{i}-x_{i}}{\alpha_{i}-\beta_{i}}+\epsilon \sum_{i=1}^{m}\lambda_{i}\frac{\alpha_{i}-x_{i}}{\alpha_{i}-\beta_{i}}$$

Given a small value of ϵ, the right term ensures that the solutions stays Pareto-optimal. More details about this method is provided in Wierzbicki (1986).

The last aggregation function tested in this study is the Ordered Weighted Averages operator (*OWA*). Through a permutation σ such that *x*_σ(1)_ ≤ *x*_σ(2)_ ≤ … ≤ *x*_σ(*m*)_ and a weighting vector *w* = (*w*_1_, …, *w*_*m*_), *w* ∈ [0, 1], the scoring function is defined as:

(23) $$owa(x)=\sum_{i=1}^{m}w_{i}x_{\sigma (i)}$$

Interestingly, varying *w* changes the behavior of the function to a minimum, maximum, or median operator. More details can be found in Yager (2004).

## Authors contributions

AB designed and conducted the experiments. AB analyzed the experimental results. GV, BG, and MK designed the computational model. GV programmed the computational model. GV, MK, BG analyzed the simulation results. GV, MK, AB, BG contributed to the writing of the manuscript.

## Funding

This work has been partly funded by the Centre National de la Recherche Scientifique PEPS Program GoHaL Project (AB, BG, MK, GV), by the Ville de Paris HABOT Project (BG, MK, GV), by the French Agence Nationale de la Recherche (ANR) Learning Under Uncertainty Project under reference ANR-11-BSV4-006 (BG, MK, GV), by Labex SMART (ANR-11-LABX-65) *Online Budgeted Learning* Projet supported by French state funds managed by the ANR within the Investissements d'Avenir programme under reference ANR-11-IDEX-0004-02, by ANR-11-IDEX-0004-02 Idex SUPER Sorbonne-Universités SU-15-R-PERSU-14 PERSU Robot Parallearning Project (MK), and by a B2V Ph.D fellowship (GV).

### Conflict of interest statement

The authors declare that the research was conducted in the absence of any commercial or financial relationships that could be construed as a potential conflict of interest.
